# Elevated White Blood Cell Count Is Associated with Higher Risk of Glucose Metabolism Disorders in Middle-Aged and Elderly Chinese People

**DOI:** 10.3390/ijerph110505497

**Published:** 2014-05-20

**Authors:** Hua Jiang, Wen-Hua Yan, Chan-Juan Li, An-Ping Wang, Jing-Tao Dou, Yi-Ming Mu

**Affiliations:** 1 Department of Endocrinology, Chinese People’s Liberation Army (PLA) General Hospital, 28 Fuxing Road, Beijing 100853, China; E-Mails: nndwmnby@gmail.com (H.J.); yanwenhua301@163.com (W.-H.Y.); anping1128@hotmail.com (A.-P.W.); jingtaodou@sohu.com (J.-T.D.); 2 Department of Endocrinology, The First Affiliated Hospital, Chinese People’s Liberation Army (PLA) General Hospital, 51 Fucheng Road, Beijing 100048, China; 3 Department of Health Statistics, Fourth Military Medical University, 169 Changle West Road, Xi’an 710032, China; E-Mail: lichanjuan@fmmu.edu.cn

**Keywords:** white blood cell, diabetes mellitus, type 2, glucose metabolism disorders, inflammation, glycosylated hemoglobin A1c

## Abstract

White blood cell (WBC) count has been associated with diabetic risk, but whether the correlation is independent of other risk factors has hardly been studied. Moreover, very few such studies with large sample sizes have been conducted in Chinese. Therefore, we investigated the relationship between WBC count and glucose metabolism in china. We also examined the relevant variables of WBC count. A total of 9,697 subjects (mean age, 58.0 ± 9.1 years) were recruited. The subjects were classified into four groups, including subjects with normal glucose tolerance, isolated impaired fasting glucose, impaired glucose tolerance and type 2 diabetes mellitus (T2DM). We found that WBC count increased as glucose metabolism disorders exacerbated. WBC count was also positively correlated with waist hip ratio, body mass index, smoking, triglycerides, glycosylated haemoglobin A1c (HbA1c) and 2-h postprandial glucose. In addition, high density lipoprotein and the female gender were inversely correlated with WBC levels. In patients with previously diagnosed T2DM, the course of T2DM was not correlated with WBC count. Our findings indicate that elevated WBC count is independently associated with worsening of glucose metabolism in middle-aged and elderly Chinese. In addition, loss of weight, smoking cessation, lipid-modifying therapies, and control of postprandial plasma glucose and HbA1c may ameliorate the chronic low-grade inflammation.

## 1. Introduction

Chronic low-grade inflammation has been shown as a key component in the pathogenesis of insulin resistance and type 2 diabetes (T2DM) [1]. Inflammation on its own can impair insulin signaling [2], and promote β-cell death [3]. Evidence from epidemiological studies suggests an association between the white blood cell (WBC) count, a non-specific marker of inflammation, and diabetes risk [4]. However, it is unknown whether the correlation is independent of other risk factors of T2DM. Moreover, this correlation remains controversial among different populations [5,6,7,8]. A review [9] suggested that the observed association was likely an over estimation. In China, the prevalence of diabetes and prediabetes are 9.7% and 15.5%, respectively, accounting for some 92.4 million adults with diabetes and 148.2 million with prediabetes [10]. A statement from the International Diabetes Federation (IDF) indicates that China has become the country with the largest number of people with diabetes. However, very few large sample size studies have been conducted to evaluate the relationship between WBC count and risk of developing diabetes in China. In this report, we examined the relationship between WBC counts and gradually worsening glucose status in consideration of known possible risk factors including age, obesity, family history of diabetes, history of hypertension, history of dyslipidemia, and history of coronary heart disease. We then explored the relevant variables that may have an effect on the WBC count.

## 2. Subjects and Methods

### 2.1. Subjects

The subjects were recruited to the study from November 2011 to August 2012. A cluster random sampling method was used in this survey. Three communities including Gucheng, Jinding street and Laoshan were randomly selected from the nine communities of Shijingshan District, Beijing’s urban area. The inclusion criteria were people of 35 years or older, who agreed to participate in the study. Patients with impaired movement, communication disability, type 1 diabetes, acute infection, and consumption of drug affecting blood WBC count, such as for hyperthyroidism, immune and hematological diseases, were excluded. All participants were permanent residents. 21,428 people were surveyed, and 19,434 people responded, with a response rate 90.69%. A random sample of 9,778 people was selected for extensive laboratory examinations. Filtered according to the inclusion criteria and exclusion criteria, a total of 9,697 subjects (3,530 males and 6,167 females) were selected for the analysis. Characteristics between the samples and the remaining people were compared and no significant difference was found with regard to age and sex. The study was approved by the ethics committee of Chinese PLA General Hospital.

### 2.2. Measurements

The participants were screened by: (a) a questionnaire for a detailed medical history, including lifestyle variables, medical history and medication use; (b) routine physical examinations that measured height, weight, blood pressure, waist circumference, and hip circumference.; (c) laboratory tests including 75 g oral glucose tolerance test (OGTT) or 100 g steamed bread test, WBC count, glycosylated hemoglobin A1c (HbA1c), and lipid profiles.

Weight and height were measured when the subjects were in light clothing without shoes. Blood pressure was measured on the right arm from a sitting position following a 5 min rest. Waist circumference was measured with a soft tape while the subject was standing, midway between the lowest rib and the iliac crest. Hip circumference was measure at the fullest part of hip a straight line around the body. After a 12 h overnight fasting, subjects without a history of diabetes mellitus underwent a 2 h 75 g OGTT. The 2-h postprandial venous blood samples were drawn from subjects with a history of diabetes after having 100 g of steamed bread. All subjects had been told to eat dinner before 8 p.m. in the day before interview, the fasting blood samples were dawn at 8–9 a.m. in the next day, and glucose water or 100 g steamed bread was taken before 9 a.m. to ensure blood could be drawn before 12 p.m..

Venous blood glucose, total cholesterol, triglyceride and high- and low-density lipoprotein cholesterol (HDL-C and LDL-C, respectively) were measured using standard enzymatic automated methods. HbA1c was determined with a High Performance Liquid Chromatography (HPLC) method [11]. WBC count was measured using an automated analyzer Sysmex XE-5000 (Sysmex Corporation, Kobe, Japan). All tests were made in the same laboratory. Quality controls were carried out daily. Researchers and laboratory personnel did not have access to identifiable information, and could only identify samples by numbers.

Based on the 75 g oral glucose tolerance test and medical history, the subjects were classified into four groups according to the 2003 American Diabetes Association (ADA) recommendations [12]: normal glucose tolerance (NGT) (fasting plasma glucose ‒ FPG) < 5.6 mmol/L and 2-h postprandial plasma glucose (2-h PG) < 7.8 mmol/L); isolated impaired fasting glucose (iIFG) (FPG 5.6–6.9 mmol/L and 2-h PG < 7.8 mmol/L); impaired glucose tolerance (IGT) (FPG < 7 mmol/L and 2-h PG 7.8–11.0 mmol/L); and T2DM (FPG ≥ 7.0 mmol/L or a 2-h PG ≥ 11.1 mmol/L). The smoking status of subjects was classified as non-smokers or smokers (including former and current smokers). Alcohol consumption status was classified as non-drinkers or drinkers (including former and current drinkers).

### 2.3. Statistical Analysis

Statistical analyses were performed using SPSS version 16.0. Data were shown as mean ± SD, unless stated otherwise. Comparisons of quantitative variables among different groups were performed by one-way ANOVA analysis. Dunnett’s T3 test was used when a significant *F* value was identified in one-way ANOVA. The chi-square test was used for categorical variables. WBC count specific risk estimates were obtained through multinomial logistic regression with iIFG, IGT or T2DM as dependent variable respectively after adjusting for age, gender, history of hypertension, family history of diabetes, history of coronary heart diabetes, history of dyslipidemia, smoking, alcohol, body mass index (BMI), and waist hip ratio (WHR). Multiple linear regression analyses were then performed to test independent associations between WBC count and the variables of interest. A *p*-value < 0.05 (two-tailed) was considered statistically significant.

## 3. Results and Discussion

### 3.1. Characteristics of Study Participants

A total of 9,697 subjects (mean age, 58.0 ± 9.1; male/female, 3,530/6,167) participated in this study. Of these, 2,486 subjects had T2DM (1,330 were previously diagnosed and 1,156 were newly diagnosed), 1,648 had iIFG, 1,889 had IGT (739 were iIGT and 1,150 were IFG/IGT), and 3,674 subjects maintained NGT. The demographic data and hematologic parameters were shown in Table 1. 

**Table 1:** Demographic data and hematologic parameters among subjects with different glycemic status.

Parameters	NGT (*n* = 3,674)	iIFG (*n* = 1,648)	IGT (*n* = 1,889)	T2DM			*P* value
				New (*n* = 1,156)	Previous (*n* = 1,330)	Total (*n* = 2,486)	
Gender (male/female)	1,158/2,516	684/964	622/1,267	473/683	593/737	1,066/1,420	<0.001
Age (years)	55.4 ± 8.4	57.2 ± 8.4	59.5 ± 9.3	60.1 ± 9.5	61.8 ± 9.2	61.0 ± 9.4	<0.001
History of hypertension (%)	22.4	29.2	37.1	43.4	54.3	49.2	<0.001
Family history of diabetes (%)	23.2	25.4	27.4	28.5	45.3	37.5	<0.001
History of coronary heart disease (%)	5.4	6.5	8.6	8.3	22.4	15.8	<0.001
History of dyslipidemia (%)	13.7	16.2	21.2	21.6	35.8	29.2	<0.001
Smoker (%)	21.7	26.3	21.4	25.2	27.2	26.3	<0.001
Drinker (%)	16.0	21.8	15.6	22.8	18.7	20.6	<0.001
WHR	0.88 ± 0.06	0.90 ± 0.06	0.91 ± 0.06	0.92 ± 0.06	0.92 ± 0.06	0.92 ± 0.06	<0.001
BMI (kg/m^2^)	25.2 ± 3.3	26.2 ± 3.4	26.7 ± 3.5	27.4 ± 6.6	26.3 ± 3.3	26.8 ± 5.1	<0.001
SBP (mmHg)	129.5 ± 30.2	133.7 ± 16.1	135.9 ± 17.1	140.3 ± 20.7	137.5 ± 17.3	138.8 ± 19.0	<0.001
DBP (mmHg)	74.8 ± 29.4	76.0 ± 9.9	75.7 ± 12.1	77.7 ± 16.8	73.1 ± 10.0	75.2 ± 13.8	0.174
WBC (cells/mm^3^)	5,794 ± 1523	6,037 ± 1,564	6,108 ± 1,504	6,532 ± 1,709	6,552 ± 1,675	6,543 ± 1,691	<0.001
Total cholesterol (mmol/L)	5.3 ± 1.4	5.4 ± 1.0	5.4 ± 1.1	5.5 ± 2.1	5.2 ± 1.1	5.3 ± 1.7	0.010
Triglycerides (mmol/L)	1.4 ± 1.1	1.6 ± 1.3	1.8 ± 1.2	2.0 ± 1.5	1.7 ± 1.4	1.8 ± 1.4	<0.001
LDL (mmol/L)	3.2 ± 0.8	3.3 ± 0.8	3.3 ± 0.8	3.4 ± 0.9	3.1 ± 0.9	3.3 ± 0.9	<0.001
HDL (mmol/L)	1.5 ± 0.4	1.4 ± 0.5	1.4 ± 0.4	1.3 ± 0.4	1.4 ± 0.4	1.3 ± 0.4	<0.001
HbA1c (%)	5.8 ± 0.4	6.0 ± 0.4	6.0 ± 0.4	6.9 ± 1.2	7.6 ± 1.6	7.3 ± 1.5	<0.001
FPG (mmol/L)	5.2 ± 0.3	6.0 ± 0.3	5.8 ± 0.5	7.5 ± 2.1	8.7 ± 2.9	8.2 ± 2.6	<0.001
2-h PG (mmol/L)	5.9 ± 1.1	6.3 ± 1.0	9.0 ± 0.9	13.7 ± 4.2	14.1 ± 4.8	13.9 ± 4.6	<0.001

Continuous data were expressed as mean ± SD. Categorical data were presented as numbers or percentages. WBC, white blood cell; NGT, normal glucose tolerance; iIFG, isolated impaired fasting glucose; IGT, impaired glucose tolerance; T2DM, type 2 diabetes mellitus; HDL, high density lipoprotein; LDL, low density lipoprotein; SBP, systolic blood pressure; DBP, diastolic blood pressure; FPG, fasting plasma glucose; 2-h PG, 2-h postprandial plasma glucose; BMI, body mass index; WHR, waist hip ratio; HbA1c, glycosylated haemoglobin A1c.

Comparing subjects of different glycemic status, we found that age, gender, history of hypertension, family history of diabetes, history of coronary heart disease, history of dyslipidemia, smoking, alcohol, WHR, BMI, systolic blood pressure (SBP), WBC count, total cholesterol, triglyceride, LDL, HDL, HbA1c, FBG and 2-h PG were significantly different among the groups (Table 1).

### 3.2. High White Blood Cell Count Is Associated With Worsening of Glucose Metabolism

**Figure 1:**
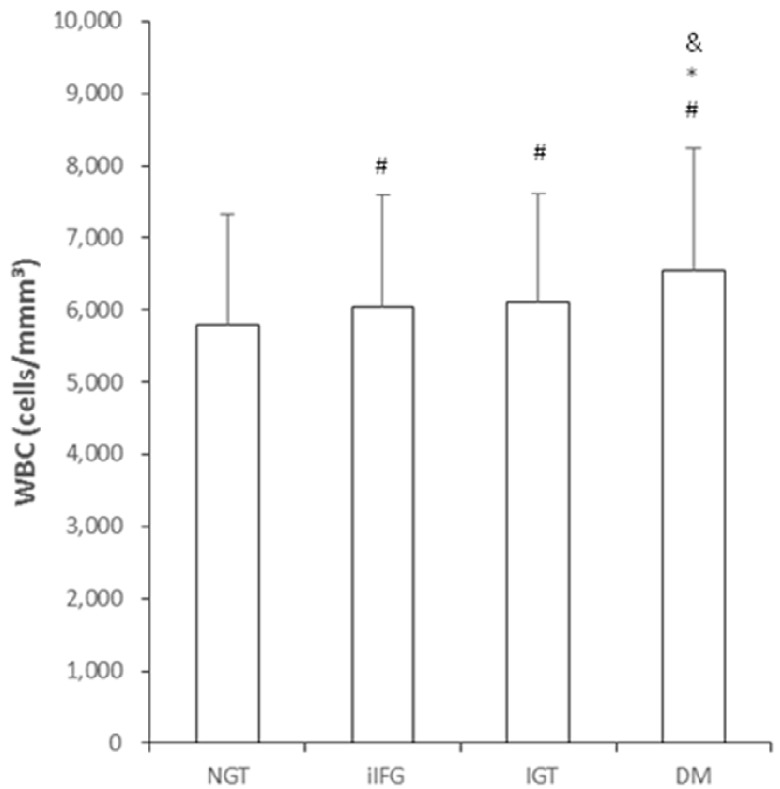
Comparisons of WBC counts between different groups. **#** indicated *P* < 0.05 compared with the NGT group. * indicated *P* < 0.05 compared with the iIFG group. & indicated *P* < 0.05 compared with the IGT group.

The mean WBC count was 6,089 ± 1,598 cells/mm^3^. By one-way ANOVA analysis, we found that WBC count increased significantly as glucose metabolism exacerbated when compared with that of NGT (*P* < 0.001, <0.001, and <0.001, respectively). No significant difference was found in the WBC counts between iIFG (6,037 ± 1,564) and iIGT (5,965 ± 1,527) (*P* = 0.967), so iIGT and IFG/IGT were combined in IGT. No significant difference was found in the WBC counts between iIFG and IGT (*P* = 0.664). The WBC count of T2DM was significantly higher than that of IGT and iIFG groups (*P* < 0.001 and <0.001, respectively) (Figure 1).

### 3.3. WBC Count may be an Independent Predictor for Developing T2DM

Many factors may affect the occurrence and development of T2DM. By multinomial logistic regression, we found that: (1) Age, history of hypertension, family history of diabetes, BMI and elevated WBC count were independent associated with iIFG. (2) Age, history of hypertension, family history of diabetes, BMI, WHR and elevated WBC count were independent associated with of IGT. (3) Age, history of hypertension, family history of diabetes, history of coronary heart disease , history of dyslipidemia, BMI, WHR and elevated WBC count were independent associated with of T2DM (Table 2). For all groups, WBC count was an independent related factor as the odds ratios were 1.094 (CI, 1.045–1.146) for iIFG, 1.122 (CI, 1.074–1.173) for IGT, and 1.300 (CI, 1.246–1.356) for T2DM.

### 3.4. Independent Relevant Variables Associated with WBC Count

Through multiple linear regression analyses, we found that WBC level was directly correlated with glycemic status, WHR, BMI, smoking, triglycerides level, HbA1c and 2-h PG; HDL and the female gender factor were inversely correlated with WBC level; Age, alcohol, BP, total cholesterol, LDL and FPG were not significantly correlated with WBC count (Table 3).

**Table 2:** Multinomial Logistic Regression Analysis with WBC Count and Possible Risk Factors as Independent Variables and Glycemic Status as Dependent Variables.

	iIFG			IGT			T2DM	
	OR	*P*		OR	*P*		OR	*P*
Age	1.025	<0.001		1.052	<0.001		1.071	<0.001
Female gender	0.801	0.05			*NS*			*NS*
History of hypertention	1.174	0.048		1.341	<0.001		1.929	<0.001
Family history of diabetes	1.231	0.011		1.557	<0.001		2.867	<0.001
History of coronary heart disease		*NS*			*NS*		1.597	<0.001
History of dyslipidemia		*NS*			*NS*		1.600	<0.001
Smoking		*NS*			*NS*			*NS*
Drinking		*NS*			*NS*			*NS*
WHR		*NS*		63.778	<0.001		228.121	<0.001
BMI	1.074	<0.001		1.087	<0.001		1.081	<0.001
WBC	1.094	<0.001		1.122	<0.001		1.300	<0.001

*NS*: the variable was not enrolled by multinomial logistic regression. OR: odds ratio.

**Table 3:** Relevant Variables associated with WBC Count in NGT, IGR and newly diagnosed DM.

		β	*P*
Grouping	NGT	0.092	0.017
	IGR		
	DM		
Age			*NS*
Female gender		−0.199	0.001
Smoking		0.568	<0.001
Drinking			*NS*
WHR		0.891	0.010
BMI		0.016	0.001
SBP (mmHg)			*NS*
DBP (mmHg)			*NS*
Total cholesterol (mmol/L)			*NS*
Triglycerides (mmol/L)		0.051	0.002
LDL (mmol/L)			*NS*
HDL (mmol/L)		−0.306	<0.001
HbA1c (%)		0.109	0.002
FPG (mmol/L)			*NS*
2-h PG (mmol/L)		0.035	<0.001

*NS*: the variable was not accepted as significant for stepwise analysis. β: unstandardized regression coefficients

Considering the course of T2DM and the use of hypoglycemic drugs, a separate linear regression model was made in previously diagnosed T2DM subjects. Compared with in subjects who were NGT, IGR and newly diagnosed T2DM, in previously diagnosed T2DM subjects, the WBC count was positively correlated with smoking, BMI and HbA1c; HDL negatively correlated with WBC count; course of T2DM, age, gender, alcohol, BP, total cholesterol, LDL, triglycerides, WHR, FPG and 2-h PG was not significantly correlated with WBC count (Table 4).

**Table 4:** Relevant Variables associated with WBC Count in previously diagnosed DM.

	β	*P*
Age		*NS*
Female gender		*NS*
Course of DM		*NS*
Smoking	0.370	0.002
Drinking		*NS*
WHR		*NS*
BMI	0.061	<0.001
SBP (mmHg)		*NS*
DBP (mmHg)		*NS*
Total cholesterol (mmol/L)		*NS*
Triglycerides (mmol/L)		*NS*
LDL (mmol/L)		*NS*
HDL (mmol/L)	−0.517	0.001
HbA1c (%)	0.079	0.013
FPG (mmol/L)		*NS*
2-h PG (mmol/L)		*NS*

*NS*: the variable was not accepted as significant for stepwise analysis. β: unstandardized regression coefficients

### 3.5. Discussion

In this study, elevated WBC count was significantly associated with the worsening of glucose metabolism (iIFG, IGT, and T2DM). This is consistent with previous studies in whites [13], Pima Indians [14], Asian Indians [15], and Japanese [16]. However, in the Cardiovascular Health Study including 4,926 [94.7%] white, 244 [4.7%] black, 31 [0.6%] other [5], no correlation of WBC count with the development of glucose metabolism disorders was found. In a study in Africa [17], it was found that WBC counts increased with increasing numbers of metabolic syndrome components in both men and women although statistical significance was not reached. Tian *et al*. [18] also found the association, but WBC counts had little difference between NGT and iIFG, or IGT and T2DM, possibly due to the smaller sample size. Furthermore, we showed that this association was present after adjustments of known possible risk factors of glucose status change (age, obesity, history of hypertension, family history of diabetes, history of dyslipidemia and history of coronary heart disease). These results suggest that elevated WBC count is an independent risk factor for IGR and T2DM. Although the mechanism underlying this increase of the WBC counts in IGR or T2DM patients remains unclear, insulin resistance might be partly responsible [14,19]. Defects in insulin action on the main insulin-sensitive tissues (adipose tissue, muscle, and liver) lead to a chronic, low-grade inflammatory state. Both intra-arterial inflammation and extra vascular stimuli can induce the secretion of proinflammatory cytokines, which promote differentiation and maturation of leukocytes. Independent of the triggering agents and the initial events, the relationship is bidirectional: any process linked to chronic inflammation will decrease insulin action, and insulin resistance will promote inflammation in a vicious cycle [20].

By multiple linear regression analyses, we found that the WBC count was correlated with not only glycemic status but also various factors. High BMI and WHR had important adverse influences. Our findings are in agreement with the results from previous large prospective cohort studies [21,22]. Chae *et al.* [23] further found that in the overweight and obese, long-term mild weight loss could reduces levels of WBC counts and inflammatory cytokine such as serum interleukin (IL)-1β, IL-6, tumor necrosis factor-α, and oxidative stress induced by inflammatory mediators.

A positive association observed in our study between the WBC count and high serum triglyceride concentrations or low HDL cholesterol components of metabolic syndrome is in agreement with previous cross-sectional studies [24,25]. It is possible that some of the lipid abnormalities observed may be a consequence of low-level inflammation [26]. Interestingly, our study found that total cholesterol and LDL were not correlated with WBC count in subjects regardless whether they have diabetes or not, which indicates that although LDL cholesterol is the primary target of cholesterol management strategies, more attention should be focused on the role of inflammation, HDL cholesterol, and triglycerides [27].

The association between smoking and WBC count was statistically significant, which was in agreement with several previous studies [28,29], Although the amount and duration of smoking were not considered in our study, smoking showed a great effect on leukocyte, so smoking cessation might be an important approach to reduce the inflammatory state. 

A positive relationship was also found between 2-h PG and WBC count, consistent with several previous studies [13,18]. The mechanism may be through the neutrophil increment during postprandial lipemia and glycemia [30] and the postprandial intravascular inflammatory changes may be relevant for the pathogenesis of atherosclerosis. We also found that HbA1c was independently correlated with the WBC counts in both subjects with or without diabetes. Few studies have addressed the possible relationships between HbA1c and WBC count in diabetes. Meanwhile the positive relationship was not found between FPG and WBC count. However, Babio *et al*. [31] revealed that fasting glucose was correlated with the WBC count. Further studies are needed to fully elucidate the mechanism. 

In this study, the female gender factor was inversely correlated with WBC level. Although the observed gender difference remains to be illustrated, the vascular protective effects of estrogen might be involved. Estrogen, a female sex hormone, is widely believed to be responsible for protection of women from atherosclerosis by decreasing the inflammatory cell adhesion as well as vasodilatation or increasing nitric oxide (NO) production [32]. Estrogen has also been shown to reduce various adhesion molecules and chemo tactic proteins that are involved in the pathogenic mechanism of atherosclerosis [33]. Hence, in our previously diagnosed T2DM subjects, the relationship between gender and WBC count was not significant partly because most of the women included in the group were old and menopausal. 

Lastly, the relation between elevated WBC count and hypertension, which has been reported in previous studies [34,35], was not observed in the present study. The result agreed with that in the African Americans and Caucasians [25]. More interestingly, course of T2DM was not significantly correlated with WBC count in previously diagnosed T2DM subjects, which has not been studied before. This may suggest that the severity of the inflammatory process of diabetes could be intervened.

This study has limitations. Firstly, the study was conducted with a cross-sectional design, making it difficult to determine causality with regard to the observed relationship. Secondly, given the potential role of insulin resistance as a mediator in the relationship between inflammation and abnormal glucose metabolism, our future studies should take this variable into account.

## 4. Conclusions

In conclusion, we found that in a large Chinese population, elevated circulating WBC count was associated with worsening of glucose metabolism even when the WBC level was within the normal range. Elevated plasma WBC count could indicate higher risk of IGR and T2DM. WBC count was also associated with WHR, BMI, smoking, triglycerides, HDL level, HbA1c and 2-h PG, suggesting that weight loss, early smoking cessation, lipid-modifying therapies, and control of postprandial plasma glucose and HbA1c may ameliorate the chronic subclinical inflammation. Understanding the role of inflammation in the development of diabetes is important for developing future prevention and treatment strategies of diabetes.

## References

[1] Donath M.Y., Shoelson S.E. (2011). Type 2 diabetes as an inflammatory disease. Nat. Rev. Immunol..

[2] Hotamisligil G.S. (2006). Inflammation and metabolic disorders. Nature.

[3] Donath M.Y., Ehses J.A., Maedler K., Schumann D.M., Ellingsgaard H., Eppler E., Reinecke M. (2005). Mechanisms of beta-cell death in type 2 diabetes. Diabetes.

[4] Twig G., Afek A., Shamiss A., Derazne E., Tzur D., Gordon B., Tirosh A. (2013). White blood cells count and incidence of type 2 diabetes in young men. Diabetes Care.

[5] Barzilay J.I., Abraham L., Heckbert S.R., Cushman M., Kuller L.H., Resnick H.E., Tracy R.P. (2001). The relation of markers of inflammation to the development of glucose disorders in the elderly: The Cardiovascular Health Study. Diabetes.

[6] Kim J.A., Choi Y.S., Hong J.I., Kim S.H., Jung H.H., Kim S.M. (2006). Association of metabolic syndrome with white blood cell subtype and red blood cells. Endocr. J..

[7] Chen W., Srinivasan S.R., Xu J., Berenson G.S. (2010). Black-white divergence in the relation of white blood cell count to metabolic syndrome in preadolescents, adolescents, and young adults: The Bogalusa Heart Study. Diabetes Care.

[8] Pratley R.E., Wilson C., Bogardus C. (1995). Relation of the white blood cell count to obesity and insulin resistance: Effect of race and gender. Obes. Res..

[9] Gkrania-Klotsas E., Ye Z., Cooper A.J., Sharp S.J., Luben R., Biggs M.L., Chen L.K., Gokulakrishnan K., Hanefeld M., Ingelsson E. (2010). Differential white blood cell count and type 2 diabetes: Systematic review and meta-analysis of cross-sectional and prospective studies. PLOS ONE.

[10] Yang W., Lu J., Weng J., Jia W., Ji L., Xiao J., Shan Z., Liu J., Tian H., Ji Q. (2010). Prevalence of diabetes among men and women in China. N. Engl. J. Med. Overseas Ed..

[11] Consensus Committee (2007). Consensus statement on the worldwide standardization of the hemoglobin A1C measurement: the American Diabetes Association, European Association for the Study of Diabetes, International Federation of Clinical Chemistry and Laboratory Medicine, and the International Diabetes Federation. Diabetes Care.

[12] The Expert Committee on the Diagnosis and Classification of Diabetes Mellitus (2003). Report of the expert committee on the diagnosis and classification of diabetes mellitus. Diabetes Care.

[13] Fritsche A., Haring H., Stumvoll M. (2004). White blood cell count as a predictor of glucose tolerance and insulin sensitivity. The role of inflammation in the pathogenesis of type 2 diabetes mellitus. Deutsche medizinische Wochenschrift (1946).

[14] Vozarova B., Weyer C., Lindsay R.S., Pratley R.E., Bogardus C., Tataranni P.A. (2002). High white blood cell count is associated with a worsening of insulin sensitivity and predicts the development of type 2 diabetes. Diabetes.

[15] Gokulakrishnan K., Deepa R., Sampathkumar R., Balasubramanyam M., Mohan V. (2009). Association of leukocyte count with varying degrees of glucose intolerance in Asian Indians: The Chennai Urban Rural Epidemiology Study (CURES-26). Metab. Syndr. Relat. Disord..

[16] Nakanishi N., Yoshida H., Matsuo Y., Suzuki K., Tatara K. (2002). White blood-cell count and the risk of impaired fasting glucose or type II diabetes in middle-aged Japanese men. Diabetologia..

[17] Nebeck K., Gelaye B., Lemma S., Berhane Y., Bekele T., Khali A., Haddis Y., Williams M.A. (2012). Hematological parameters and metabolic syndrome: Findings from an occupational cohort in Ethiopia. Diabetes Metab. Syndr..

[18] Tian J.Y., Yang Y., Cheng Q., Huang H.E., Li R., Jiang G.X., Liu S.Y., Li X.Y., Ning G. (2008). Association of WBC count and glucose metabolism among Chinese population aged 40 years and over. Diabetes Res. Clin. Pract..

[19] Chen L.K., Lin M.H., Chen Z.J., Hwang S.J., Chiou S.T. (2006). Association of insulin resistance and hematologic parameters: Study of a middle-aged and elderly Chinese population in Taiwan. J. Chin. Med. Assoc..

[20] de Luca C., Olefsky J.M. (2008). Inflammation and insulin resistance. FEBS Lett..

[21] Meng W., Zhang C., Zhang Q., Song X., Lin H., Zhang D., Zhang Y., Zhu Z., Wu S., Liu Y. (2012). Association between leukocyte and metabolic syndrome in urban han Chinese: a longitudinal cohort study. PLOS ONE.

[22] Holz T., Thorand B., Doring A., Schneider A., Meisinger C., Koenig W. (2010). Markers of inflammation and weight change in middle-aged adults: Results from the prospective MONICA/KORA S3/F3 study. Obesity (Silver Spring, Md.).

[23] Chae J.S., Paik J.K., Kang R., Kim M., Choi Y., Lee S.H., Lee J.H. (2013). Mild weight loss reduces inflammatory cytokines, leukocyte count, and oxidative stress in overweight and moderately obese participants treated for 3 years with dietary modification. Nutr. Res. (New York, N.Y.).

[24] Huang Z.S., Chien K.L., Yang C.Y., Tsai K.S., Wang C.H. (2001). Peripheral differential leukocyte counts in humans vary with hyperlipidemia, smoking, and body mass index. Lipids.

[25] Boucher A.A., Edeoga C., Ebenibo S., Wan J., Dagogo-Jack S. (2012). Leukocyte Count and cardiometabolic risk among healthy participants with parental type 2 diabetes: The pathobiology of prediabetes in a biracial cohort study. Ethn. Dis..

[26] Khovidhunkit W., Kim M.S., Memon R.A., Shigenaga J.K., Moser A.H., Feingold K.R., Grunfeld C. (2004). Effects of infection and inflammation on lipid and lipoprotein metabolism: Mechanisms and consequences to the host. J. Lipid Res..

[27] Gotto A.M., Moon J.E. (2012). Recent clinical studies of the effects of lipid-modifying therapies. Am. J. Cardiol..

[28] Ishizaka N., Ishizaka Y., Toda E., Nagai R., Yamakado M. (2007). Association between cigarette smoking, white blood cell count, and metabolic syndrome as defined by the Japanese criteria. Int. Med. (Tokyo, Japan).

[29] Asthana A., Johnson H.M., Piper M.E., Fiore M.C., Baker T.B., Stein J.H. (2010). Effects of smoking intensity and cessation on inflammatory markers in a large cohort of active smokers. Am. Heart J..

[30] van Oostrom A.J., Sijmonsma T.P., Verseyden C., Jansen E.H., de Koning E.J., Rabelink T.J., Castro C.M. (2003). Postprandial recruitment of neutrophils may contribute to endothelial dysfunction. J. Lipid Res..

[31] Babio N., Ibarrola-Jurado N., Bullo M., Martinez-Gonzalez M.A., Warnberg J., Salaverria I., Ortega-Calvo M., Estruch R., Serra-Majem L., Covas M.I. (2013). White blood cell counts as risk markers of developing metabolic syndrome and its components in the PREDIMED Study. PLOS ONE.

[32] Mendelsohn M.E., Karas R.H. (2005). Molecular and cellular basis of cardiovascular gender differences. Science (New York, N.Y.).

[33] Caulin-Glaser T., Watson C.A., Pardi R., Bender J.R. (1996). Effects of 17beta-estradiol on cytokine-induced endothelial cell adhesion molecule expression. J. Clin. Invest..

[34] Shankar A., Klein B.E., Klein R. (2004). Relationship between white blood cell count and incident hypertension. Am. J. Hypertens..

[35] Friedman G.D., Selby J.V., Quesenberry C.P. (1990). The leukocyte count: A predictor of hypertension. J. Clin. Epidemiol..

